# A Two-Phase Feature Selection Method for Identifying Influential Spreaders of Disease Epidemics in Complex Networks

**DOI:** 10.3390/e25071068

**Published:** 2023-07-15

**Authors:** Xiya Wang, Yuexing Han, Bing Wang

**Affiliations:** 1 School of Computer Engineering and Science, Shanghai University, Shanghai 200444, China; sia_wong@shu.edu.cn; 2 Zhejiang Laboratory, Hangzhou 311100, China

**Keywords:** network epidemiology, influential node identification, feature selection, machine learning

## Abstract

Network epidemiology plays a fundamental role in understanding the relationship between network structure and epidemic dynamics, among which identifying influential spreaders is especially important. Most previous studies aim to propose a centrality measure based on network topology to reflect the influence of spreaders, which manifest limited universality. Machine learning enhances the identification of influential spreaders by combining multiple centralities. However, several centrality measures utilized in machine learning methods, such as closeness centrality, exhibit high computational complexity when confronted with large network sizes. Here, we propose a two-phase feature selection method for identifying influential spreaders with a reduced feature dimension. Depending on the definition of influential spreaders, we obtain the optimal feature combination for different synthetic networks. Our results demonstrate that when the datasets are mildly or moderately imbalanced, for Barabasi–Albert (BA) scale-free networks, the centralities’ combination with the two-hop neighborhood is fundamental, and for Erdős–Rényi (ER) random graphs, the centralities’ combination with the degree centrality is essential. Meanwhile, for Watts–Strogatz (WS) small world networks, feature selection is unnecessary. We also conduct experiments on real-world networks, and the features selected display a high similarity with synthetic networks. Our method provides a new path for identifying superspreaders for the control of epidemics.

## 1. Introduction

Epidemics always threaten human health and affect social stability, especially recently, in the COVID-19 pandemic. Since pandemics severely disrupt people’s daily lives, research on mitigating the impacts of the disease is attracting more and more attention. Moreover, based on the promising performance of machine learning, studies exploring pandemics using machine learning have been emphasized in the past few years. These studies explore different types of data, including text data such as the blood test reports of patients [1]; voice data such as cough sounds [2]; image data such as X-rays, CT, and ultrasound scans [3]; and multimode data [4]. Besides the above research on clinical diagnosis, identifying influential individuals in the process of the epidemic’s spread is also an effective instrument to control epidemic propagation.

Theoretical epidemiology [5] describes the spreading rule of an epidemic as a quantitative mathematics model. Later, the complex network theory provided a new direction for spreading dynamics, and the transmission of infectious diseases in real systems can be abstracted into a dynamical process on complex networks, which is graph data. With respect to network epidemiology, the influence of each node depends on the expected outbreak size when the spread of the disease originates from the node itself.

However, it is challenging to directly estimate the influence of each individual spreader. In previous studies, traditional centrality methods [6,7] focus on a network’s structural properties and use top ranked nodes by different centralities as influential spreaders. In general, centralities can be classified as local and global measures [8]. Local measures are skewed towards the information of a neighborhood, such as degree centrality [9]. On the other hand, global measures mainly depict the position of a node in the network. For instance, closeness centrality [9] measures a spreader by averaging the shortest paths between the spreader and others, while betweenness centrality [9] is concerned with the frequency of the shortest paths between the node pair passing through the spreader. K-Shell [10] and its extension methods aim to decompose the network into different levels. Moreover, eigenvector centrality [11] is based on iteration; its variants include Katz centrality [12], PageRank [13], etc.

**Figure 1:**
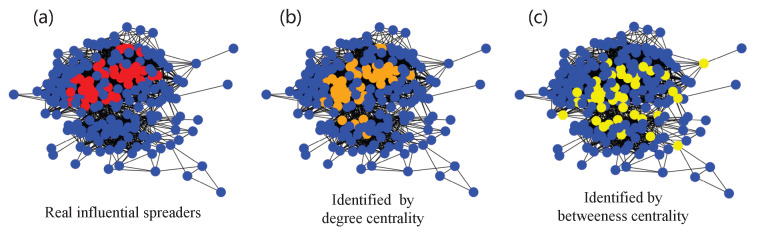
Identifying the top 20% influential spreaders in the human social network Jazz. (**a**) Real influential spreaders (red nodes) ranked by simulation results using Susceptible–Infectious–Recovered (SIR) dynamics. (**b**) Influential spreaders (orange nodes) ranked by degree centrality. (**c**) Influential spreaders (yellow nodes) ranked by betweenness centrality.

However, at present, different centrality methods describe structural properties from different aspects; thus, the definition of centrality has no uniform standard. In addition, a single centrality usually contains limited topological knowledge, resulting in an inability to completely reflect the real spreading potential of each spreader. For instance, as shown in Figure 1, neither the identification results of the degree centrality (Figure 1b) nor the betweenness centrality (Figure 1c) can cover the real situation.

To overcome the deficiencies of traditional centrality methods, machine learning has been introduced as a new tool that acquires the hidden rules from existing data and accomplishes the prediction automatically [14]. In the field of network epidemiology, since the coupling relationship between the underlying network topology and dynamic results is hard to express explicitly, machine learning provides a key to approximating it. Recently, there have been some interdisciplinary works on integrating network epidemiology and machine learning that provide a new way to solve challenges, such as epidemic threshold identification [15,16], basic reproduction number prediction [17], source tracing [18], state transition probability estimation [19], and individual’s health state inferences [20].

In relation to the identification of influential spreaders, machine learning methods use structural attributes as the input, and tasks are briefly categorized as regression and imbalanced classifications. The former task is concerned with how to predict the size of the epidemic outbreak. For example, Rodrigues et al. adopted an artificial neural network (ANN) and random forest (RF) to predict disease dynamic variables and the importance of central attributes of patient zero [21]. Bucur et al. utilized support vector machine (SVM) and RF to estimate the exact expected outbreak size calculated on all possible infection paths. They found that a spectral-based centrality plus a property based on the edge density is sufficient to accomplish the task [22]. Regarding the imbalanced classification problem, Zhao et al. defined a machine learning process to distinguish the infection capability of a spreader. By comparing the performance of seven classifiers, they demonstrated the effectiveness and the scalability of classifiers [23]. Bucur found that the combination of a local centrality and a global centrality is capable of drawing a decision boundary, which distinguishes superspreaders from others based on SVM [24]. Recently, deep learning has become an active research orientation in the field of machine learning. Yu et al. generated a feature matrix based on a adjacency matrix and the node’s degree and solved the regression problem using a convolutional neural network (CNN) [25]. Zhao et al. proposed the learning model, i.e., InfGCN based on a graph convolutional network (GCN), and classified the minority of influential spreaders from the majority of less influential spreaders [26].

The machine learning methods that integrate multiple centrality methods, are more predictive than a single centrality method. However, these authors subjectively chose some centralities as features. For instance, Zhao et al. used the combination of degree [9], betweenness centrality [9], closeness centrality [9], and clustering coefficient [27] as their features [26], which were chosen without a specific criterion. Moreover, with the growing network size, some of the centralities chosen, such as betweenness centrality and closeness centrality [9], become computationally expensive, i.e., $O({\left|V\right|}^{3})$, and they are useless for the identification task [22]. Therefore, in order to enhance efficiency, it is crucial to figure out the optimal combination of centralities for identifying influential spreaders.

Previous studies solve the insufficiency of the machine learning methods using an exhaustive search method [22,24], which also brings high computational complexity and ignores the relationship between local or global centralities. Instead of completing searches between pairs of features, feature selection methods help to reduce feature redundancy and optimize feature combination. Broadly, feature selection methods are categorized into three classes, i.e., filter, wrapper, and embedding methods [28]. The filter method is independent of the subsequent machine learning model, as it only scores features based on their own properties or the correlation between the labels and the features [29]. In contrast, the wrapper method and the embedding method involve a machine learning model [29]. The wrapper method regards the performance of classifiers like SVM as a feature subset evaluation measure. To enhance feature selection efficiency, some wrapper methods employ intelligent algorithms such as particle swarm optimization (PSO) as their feature search strategy [30,31]. The embedding method obtains the importance of features while performing the classification task, such as RF. Based on the feature selection methods mentioned above, hybrid feature selection methods and ensemble feature selection methods have achieved significant developments [28]. In order to enhance performance, hybrid feature selection methods combine various types of feature selection methods, while ensemble feature selection methods deal with the instability of feature selection. However, these methods mentioned above are not appropriate for imbalanced data, the feature subsets selected by them are biased towards the majority class, affecting the reliability of classification performance over the minority class [32].

In all, a single heuristic centrality lacks prediction capability, and machine learning methods manually choose a number of centralities as features without a consistent standard, resulting in the inclusion of redundant, computationally expensive features. In order to adaptively select important centralities, here, we propose a two-phase feature selection method for identifying influential spreaders. In view of the imbalanced data distribution, to gain the optimal combination of centralities, the initial selection is conducted using an ensemble feature selection, with sampling technology and the wrapper technique used to balance the data. To obtain representative centralities, the secondary selection adopts hierarchical clustering [33] and the filter technique. By utilizing our feature selection method on three types of synthetic networks, we find that the combination of centralities involving a two-hop neighborhood with PageRank or degree is more effective for identifying influential spreaders in BA networks, while the combination of degree and one-hop neighborhood or two-hop neighborhood centralities is key for ER networks. The performance of classifiers with our feature selection method is competitive, while the complexity of calculation is remarkably reduced. For WS networks, the identification task calls for the maximal incorporation of features, rendering the selection of features unnecessary. We also validate our model on a real-world network, which helps us to figure out the most important feature for each network.

The rest paper is organized as follows. Section 2 presents our method, including the generation of features and labels and the feature selection method. In Section 3, we show the performance of our method, comparing it with other baselines. We conclude our work in Section 4 and discuss the future work in Section 5.

## 2. Methods

**Figure 2:**
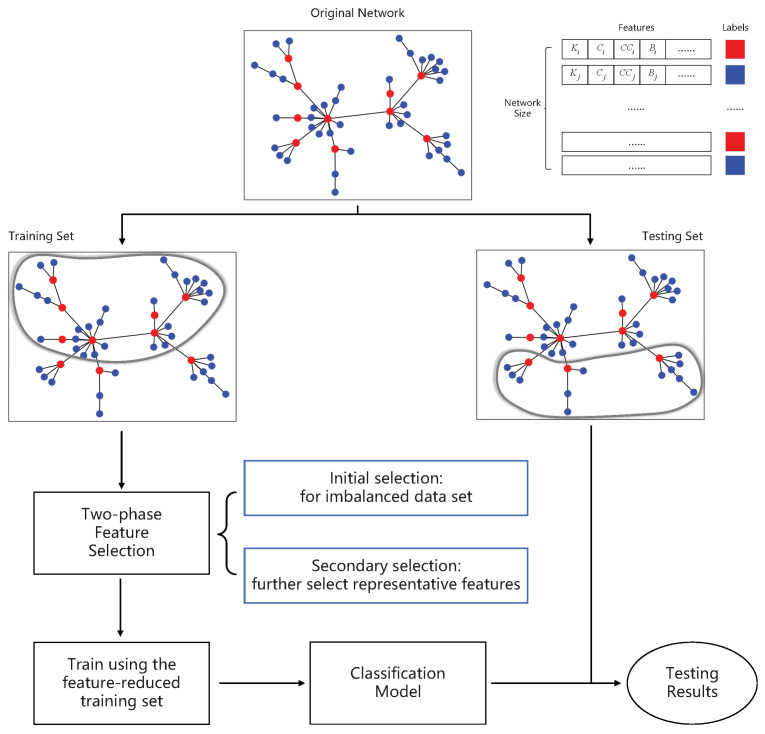
The machine learning scheme for identifying influential spreaders for disease dynamics. In the original network, the color of the node represents its spreading capability. Red nodes are influential spreaders while blue nodes are less influential spreaders.

In this section, we introduce our machine learning scheme including the proposed feature selection method for identifying influential spreaders for disease dynamics, as shown in Figure 2. It illustrates two components, i.e., the description of data (original network and the content of the data set on its right in Figure 2) and the process of training and testing. First of all, it is indispensable to construct data sets, which are the resources of machine learning models. Given a network, we choose centralities that reflect structural characteristics as features, and we label nodes as superspreaders or not according to the results of the disease dynamics in the network. Then, during the process of training, in view of high computational complexity of some centralities such as betweenness centrality, we propose a two-phase feature selection method, which reduces the feature dimensions and maintains the high performance of the classifier. After obtaining the selected features, we feed them into a classifier for the training set and conduct tests on the corresponding testing set.

### 2.1. Data Set Generation

#### 2.1.1. Features Based on Centralities

For disease dynamics, the topological attributes of the source of infection affect the spreading results. We associate the infection capability of a spreader with classical centrality measures, which characterize the network structure from various perspectives. For a network *G* with *N* nodes, the feature vector of node *i* is expressed as $X_{i}=\left[X_{i1},X_{i2},\ldots ,X_{id}\right]$, i=1,…,N, where *d* is the amount of original features obtained from different centrality methods. Here, inspired by previous works, we select 9 typical centrality methods as follows: (1) neighborhood-based centrality, including degree (*K*) [9], one-hop neighborhood ($K_{sum}$) [34], two-hop neighborhood ($K_{2sum}$) [34], K-shell (KS) [10], and clustering coefficient (*C*) [27]; (2) path-based centrality, including betweenness centrality (*B*) [9] and closeness centrality (CC) [9]; and (3) spectral-based centrality, including eigenvector centrality (EC) [11] and PageRank (PR) [13], as shown in Table 1. Among the above centralities, neighborhood-based centralities, except KS, are local measures, while path-based centralities and spectral-based centralities are global measures.

#### 2.1.2. Labels Based on the SIR Model

In network epidemiology, it is always assumed that the time scale of the network’s evolution is much larger than the epidemic dynamics; thus, the underlying network is static. During the spreading process, the influence of each spreader is measured by the outbreak size when it is taken as a seed. In order to obtain the outbreak size caused by each seed spreader, we adopt the SIR compartmental model due to its ability to reach an absorbing state, i.e., the number of infected individuals will be reduced to zero within a finite time. In addition to the field of disease dynamics, the SIR model is also utilized in rumor spreading, information spreading, etc. [6]. The SIR model defines three states, susceptible, infectious, and recovered. A node can change its state according to transition probabilities until the system reaches a steady state. At every time step *t*, each infected node transmits the disease to all its susceptible neighbors at an infection rate β and recovers at a recovery rate μ. The effective infection rate for the disease dynamics is defined as λ=β/μ. Here, we assume μ to be a fixed value for simplicity, i.e., μ=1. The epidemic threshold $\lambda_{c}$ is the vital value which distinguishes between whether a disease outbreak will or will not occur in the system. Here, $\lambda_{c}$ is numerically estimated by the variability measure as follows [35]:

(1) $$\Delta =\frac{\sqrt{\langle \rho^{2}\rangle -{\langle \rho \rangle }^{2}}}{\langle \rho \rangle },$$

where ρ is the fraction of infected nodes obtained from the SIR process caused by a random seed node. The dynamic observable ρ remains constant when the system reaches a stationary state, given by

(2) $$\rho =\lim_{t\to \infty }\frac{1}{N}\sum_{i=1}^{N}a_{i}\left(t\right),$$

where $a_{i}$ denotes the binary dynamical state of node *i*. Moreover, $a_{i}=1$ means node *i* is infected, while $a_{i}=0$ means node *i* is susceptible. In the finite range of λ, the variability measure Δ achieves the maximum at the epidemic threshold $\lambda_{c}$.

Let us take the issue of identifying the influential spreaders of a network as a classification problem. The influence of each node is labeled by $Y_{i},i=1,\ldots ,N$ according to the result of the SIR model for a given λ. More specifically, in order to quantify the infection ability of each node, initially, we successively set node i,i=1,…,N as seeded nodes, while others are susceptible. Then, the SIR process in the networks is simulated using the Monte Carlo method. We repeat the simulation $10^{3}$ times, and measure the average spreading result of node *i* for a given transmission rate λ, $\langle \rho_{i}^{\lambda }\rangle ,i=1,\ldots ,N$. Considering that the result is fine-grained, we sort $\langle \rho_{i}^{\lambda }\rangle$ in descending order. Let us define the top *f* percentage of nodes as influential spreaders, and the remaining 1−f are not; if node *i* is grouped into the top *f* spreaders, it is labeled with $Y_{i}=1$; otherwise, it is given the label $Y_{i}=-1$. The task is to classify the nodes in the network as influential or not. Table 2 gives a sample of the data set.

### 2.2. A Two-Phase Feature Selection Method

In view of the high time complexities of some of the centrality methods fed into the classifier, it is necessary to optimize feature combination. Different from previous works using exhaustive search methods, how to apply feature selection to manage the task of identifying influential spreaders is the point of our study. More specifically, according to the imbalanced data set, we propose a two-phase feature selection method in the training process, which aims to explore the best combination of features from spreading data. The process of our feature selection includes 2 phases; that is, the initial selection of our method focuses on dealing with the imbalanced data sets, and the secondary selection of our method concentrates on selecting more representative features from the results of the initial selection.

#### 2.2.1. Initial Selection of the Features

**Figure 3:**
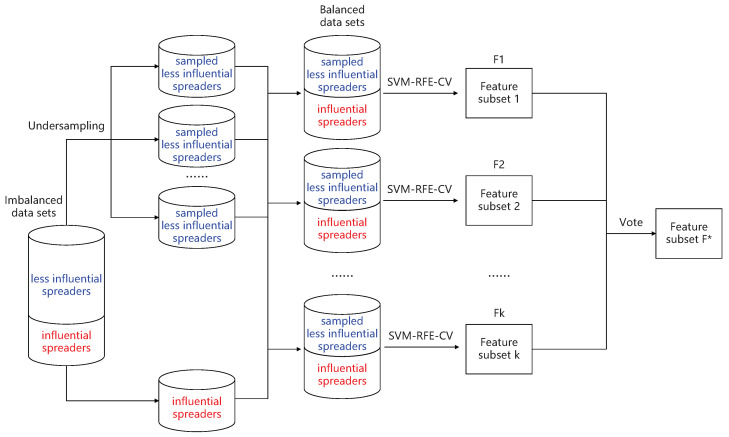
The initial selection of feature selection process. The red-colored words represent influential spreaders, while the blue-colored words represent less influential spreaders.

Considering the imbalanced data distribution of the spreading model, as shown in Figure 3, we implement the initial selection to achieve feature selection in imbalanced data sets. The initial selection is an ensemble feature selection using sampling technology and the wrapper technique [28]. We apply bootstrap sampling to balance the data sets, i.e., we randomly sample instances from the majority class until the number of sampled data is equal to that of minority class. The new balanced training data set contains both sampled less influential spreaders and unsampled influential spreaders.

Then, we need to select features on the processed balanced data sets. Considering the versatility and scalability of the SVM, we employ the wrapper technique and an SVM based on recursive feature elimination and cross-validation (SVM-RFE-CV) [36], a feature selection method that takes the results of the classifier as its evaluation criteria, which eliminates features with low weight in turn. It follows 4 steps:

- Step 1: Train SVM classifiers on the training data set with 10-fold cross-validation;
- Step 2: Summarize the 10 importance scores of each feature independently, and then accumulate the classifier performance of each fold;
- Step 3: Remove the least important feature;
- Step 4: Return to Step 1 until all features are eliminated.

The best feature subset has the highest classifier performance. We set a linear SVM with a regularization parameter as the classifier and choose weight as the importance score for each feature. Inspired by ensemble learning, we execute SVM-RFE-CV on *k* different balanced training data sets and obtain *k* feature subsets $\left\{F_{1},F_{2},\ldots ,F_{k}\right\}$. Then, we calculate the frequency of each feature over all the feature subsets by making a vote on the feature subsets. If the frequency goes beyond the given threshold ϵ, we add the feature to the subset after the initial feature selection $F^{*}$. Above all, the initial selection fully taps the imbalanced spreading data and improves the stability of the feature selection method based on the diversity of feature subsets.

#### 2.2.2. Secondary Selection of the Features

**Figure 4:**
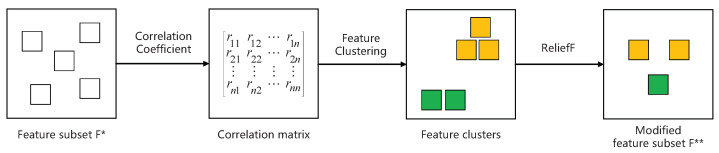
The secondary selection of the feature selection process. The colors represent the different clusters to which the features belong.

In addition, considering that the initial selection is unable to remove redundant features, to obtain representative features of different levels structure information, we further propose performing the secondary selection using hierarchical clustering and the filter technique. Figure 4 illustrates the entire process.

Determined by the feature subset $F^{*}$ resulting from the initial selection, to measure the similarity of features, we first obtain a correlation matrix of these features; more specifically, we calculate the Pearson correlation coefficient to measure the linear association between each pair of features *i* and *j*, $r_{ij}$, given by

(3) $$r_{ij}=\frac{\sum_{m}\left(x_{im}-\bar{x_{i}}\right)\left(x_{jm}-\bar{x_{j}}\right)}{\sqrt{\sum_{m}{\left(x_{im}-\bar{x_{i}}\right)}^{2}}\sqrt{\sum_{m}{\left(x_{jm}-\bar{x_{j}}\right)}^{2}}},$$

where $x_{im}$ represents the $m^{th}$ value of feature *i*; $\bar{x_{i}}$ means the average of $x_{i}$; and $r_{ij}$ is in the range of [−1,1]. A larger $|r_{ij}|$ means a higher redundancy between feature *i* and feature *j*.

Then, we apply hierarchical clustering to realize feature clustering. To be specific, initially, every single feature is regard as a cluster. Thereafter, according to the correlation matrix, we merge clusters with the largest $|r_{ij}|$ at each step. Since the previous work [22,24] sorted features into two categories and found that at least 2 features can identify top spreaders, here, we assume the number of final feature clusters to be two.

Eventually, for each cluster, we generate the final feature subset $F^{**}$ using the a filter technique, namely, the ReliefF algorithm [37], which measures the discrimination between the different classes of each feature. Another important point is the critical amount of the final selected features. Here, we define it as $\gamma =\frac{|F^{*}|}{2}$. If the number of features of a cluster is larger than γ, we select a main feature and a supplementary feature; otherwise, we only obtain a main feature. Depending on the ReliefF algorithm, we take the feature with larger weight as the main feature of the cluster, and the supplementary feature has a minimum linear association with the main feature, as evaluated by the correlation matrix. Algorithm 1 shows the pseudocode of our proposed method.
**Algorithm 1:** The proposed two-phase feature selection method FFS-SFS
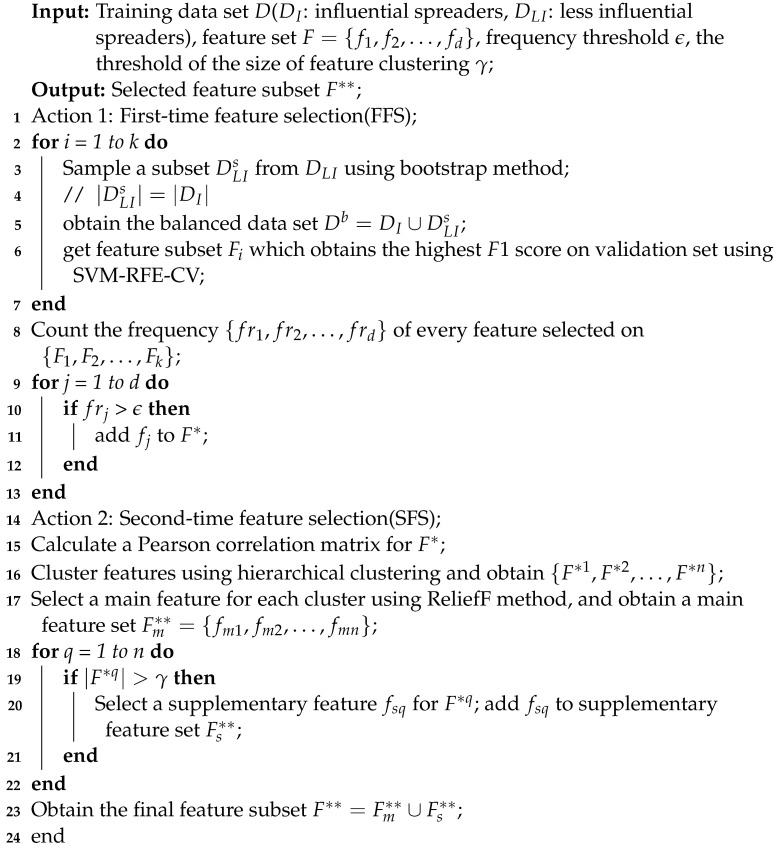


## 3. Results

In view of the coupling relationship between the network structure and the disease dynamics in networks, we mainly explore the optimal combination of centralities for various network models with the proposed two-phase feature selection method. Here, we study three representative types of synthetic networks, including BA scale-free networks [38], ER random graphs [39], and WS small world networks [40]. The network size is N=1000 and the average degree is 〈k〉=6. For each network model, we sample 10 networks. Finally, we also verify our method on real-world networks.

Since networks are assumed not to evolve over time, once the underlying network structure is fixed, the topological properties of each node remain invariant. We simulate the disease dynamics on the network and label each node *i* according to the average epidemic prevalence $\langle \rho_{i}^{\lambda }\rangle$ for a given effective infection rate λ and the percentage of influential spreaders *f*. To explore different epidemic behaviors at different infection rates, we select three representative values, i.e., $\lambda =0.5\lambda_{c}$, $\lambda_{c}$, $1.5\lambda_{c}$. Similarly, to explore the impact of the imbalanced data set, we choose f=5%, 10%, 15%, and 20%.

The data set is randomly split into two parts, 70% for training and 30% for testing. We repeat the process 30 times on each data set and obtain 30 results for each data set. Then, we select the one with the highest frequency as the final feature subset of the network. By summarizing the results of the networks generated from the same network model, we vote to select the optimal feature subset of the network model.

We systematically investigate the effects of the network model, the effective infection rate λ, and the percentage of influential spreaders *f* on the performance of the proposed feature selection method. Here, we evaluate the performance based on the number of features and the Precision, Recall, and *F*1-measure obtained on the testing sets. The performance metrics are based on a confusion matrix. Precision measures the proportion of true positives (TP) among the positive predictions, given by

(4) $$Precision=\frac{TP}{TP+FP},$$

Recall measures the ratio of the positive samples correctly identified, expressed as

(5) $$Recall=\frac{TP}{TP+FN},$$

*F*1-measure (F1) balances Precision and Recall and is written as

(6) $$F1=\frac{2}{Precision^{-1}+Recall^{-1}}.$$

To verify the effectiveness of our proposed method, we compare our method, First-time Feature Selection–Second-time Feature Selection (FFS-SFS), with 13 comparison methods, including nine single centralities (degree, one-hop, two-hop, K-Shell, clusteringcoefficient, betweenness, closeness, eigenvectorcentrality, PageRank) and four feature selection methods as follows:

(1) All: ignore feature selection and use the original feature set;
(2) Imbalanced: directly adopt SVM-RFE-CV on the raw imbalanced training set;
(3) First-time Feature Selection–ReliefF (FFS-ReliefF): filter features using ReliefF based on the result of the initial selection;
(4) First-time Feature Selection–Weight (FFS-Weight): choose features according to the frequency based on the results of the initial selection.

Both FFS-ReliefF and FFS-Weight do not include feature clustering. Additionally, they are set to the same amount of features as our selected method, and the difference between them is the selection criterion in the second phase.

Moreover, we also conduct an ablation experiment to verify the effectiveness of each phase in our feature selection method, i.e., without (w/o) SFS, which only utilizes the initial selection of our feature selection method, and w/o FFS, which only utilizes the secondary selection of our feature selection method.

### 3.1. Results on BA Networks

Firstly, we focus on the results using our feature selection method on BA networks [38], where the degree distribution is heterogeneous, i.e., there are only a small number of hub nodes in the network. Here, we conduct the experiment on BA networks with 1000 nodes and 2991 edges, and the density of the networks is 0.005988.

#### 3.1.1. Contrasting Experiment

Table 3 depicts the size of the feature subset selected by five feature selection methods among 12 configurations (the combination of different λ and *f*) and the features selected by our method. The original full set *F* includes nine centralities: degree, one-hop, two-hop, K-Shell, clusteringcoefficient, betweenness, closeness, eigenvectorcentrality, and PageRank. Since each node in the BA network obtains the same K-Shell value, i.e., KS=3, which is inadequate to identify the spreading potential, the number of centralities in the full set in Table 3 is eight.

Regarding the number of selected features (see Table 3), Imbalanced almost selects all the features, which reveals that Imbalanced has a disadvantage in the reduction in feature dimensions on the imbalanced data set. On the contrary, our method FFS-SFS can effectively decrease the number of features by about 75% in most scenarios with different epidemic parameters, where both the initial selection and the clustering module help to filter features. Although FFS-ReliefF and FFS-Weight also reduce the number of features, their processes for determining the number of selected features require manual intervention.

**Figure 5:**
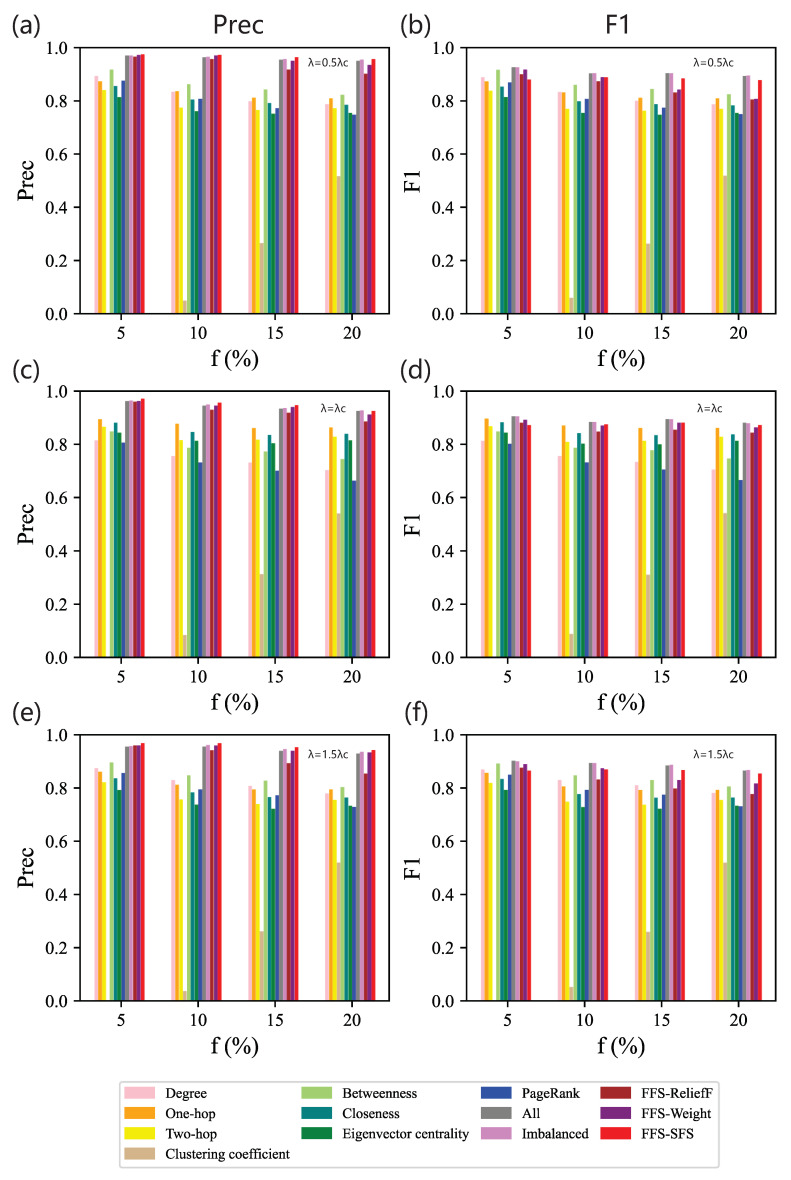
Contrasting experiments on BA networks. Precision (**left** column) and F1 (**right** column) of classifiers based on different feature subsets obtained from 13 methods on data sets for different combinations of *f* and λ: (**a**,**b**) $\lambda =0.5\lambda_{c}$; (**c**,**d**) $\lambda =\lambda_{c}$; (**e**,**f**) $\lambda =1.5\lambda_{c}$.

In order to verify the effectiveness of our method, we compare our method with eight single centralities and four feature selection methods. Figure 5 illustrates Precision and F1 of classifiers fed distinct optimal feature subsets. As shown in Figure 5a,b, when the effective infection rate is $\lambda =0.5\lambda_{c}$, a single centrality (the first eight bars), betweenness, performs better than other centralities across the whole range of *f*, while feature combinations (other bars) show better performance than a single centrality. This is because additional centralities bring more topological knowledge. In detail, the full feature set always performs the best. The result of Imbalanced is the full feature set whatever *f* we tested, so its performance is quite close to that of the full feature set, indicating that Imbalanced is insensitive to the imbalanced data distribution. While FFS-ReliefF, FFS-Weight, and FFS-SFS all depend on the initial selection, the difference among them is the criteria for the second phase of feature selection. Although their performance declines little, the dimensions of the features are dramatically reduced. Compared with FFS-ReliefF and FFS-Weight, our method FFS-SFS performs better in general. We find that feature clustering is beneficial for selecting features for imbalanced data classification. For FFS-Weight, it also shows that choosing the features with the highest weight given by the classifier is not necessarily optimal.

As for our feature selection method, FFS-SFS, we observe that it can effectively reduce the feature dimensions and meanwhile show great performance. Its Precision is superior to other methods within the assigned range of *f*. Obviously, when the data set is moderately imbalanced, i.e., *f* = 10% or 15%, Precision of our method is 1% higher than the full feature set. As for F1, though there is a narrow margin between the full feature set and the one obtained with our method, the size of the feature set selected by our method decreases significantly. Specifically, when *f* is 15%, F1 of our method only declines by 2% compared with the baseline. However, when the data set is extremely imbalanced, i.e., f=5%, F1 of our method is worse. The reason is that undersampling in the initial selection performs better on the less imbalanced data set. The initial selection of our method becomes volatile and introduces redundant features, resulting in errors in the process of feature clustering. Moreover, F1 of betweenness performs similar to the full feature set.

On the whole, when λ is fixed, with the increase of *f*, Precision and F1 of the proposed feature selection methods gradually decrease, as shown in Figure 5a,b. It may be caused by the confusion of handmade labels, which regards the real less influential spreaders as the fake influential spreaders.

When $\lambda =\lambda_{c}$, the feature combination method still performs better than the use of a single centrality, as shown in Figure 5c,d. Among the eight single centralities, one-hop discriminates spreaders with the greatest efficiency. The performance of our feature combination is better than other combination methods measured by Precision across all the ranges of *f* and is close to that of the full feature set measured by F1, which is similar to the results for $\lambda =0.5\lambda_{c}$. In addition, compared with $\lambda =0.5\lambda_{c}$, F1 scores show that our method has a slight advantage over FFS-Weight, as it improved at least 4% for $\lambda =0.5\lambda_{c}$ and 0.1% for $\lambda =\lambda_{c}$. At $\lambda =\lambda_{c}$, due to the fluctuation in the data, with greater knowledge on network topology, the gap in performance becomes smaller.

When $\lambda =1.5\lambda_{c}$, the result is similar to the one with $\lambda =0.5\lambda_{c}$, as shown in Figure 5e,f. Betweenness is the best classifier among all the single centralities, especially when the data set is extremely imbalanced, i.e., f=5%. It even defeats some feature combination methods. The feature subset selected by our method still steadily classifies spreaders and improves precision.

#### 3.1.2. Ablation Experiment

Table 4 shows the sizes of the feature subset selected by FFS, SFS, and our method, FFS-SFS. We demonstrate that FFS (our method w/o SFS), which only executes the initial selection, can effectively decrease about 50% of the features in most scenarios for different combinations of parameters λ and *f*.

**Figure 6:**
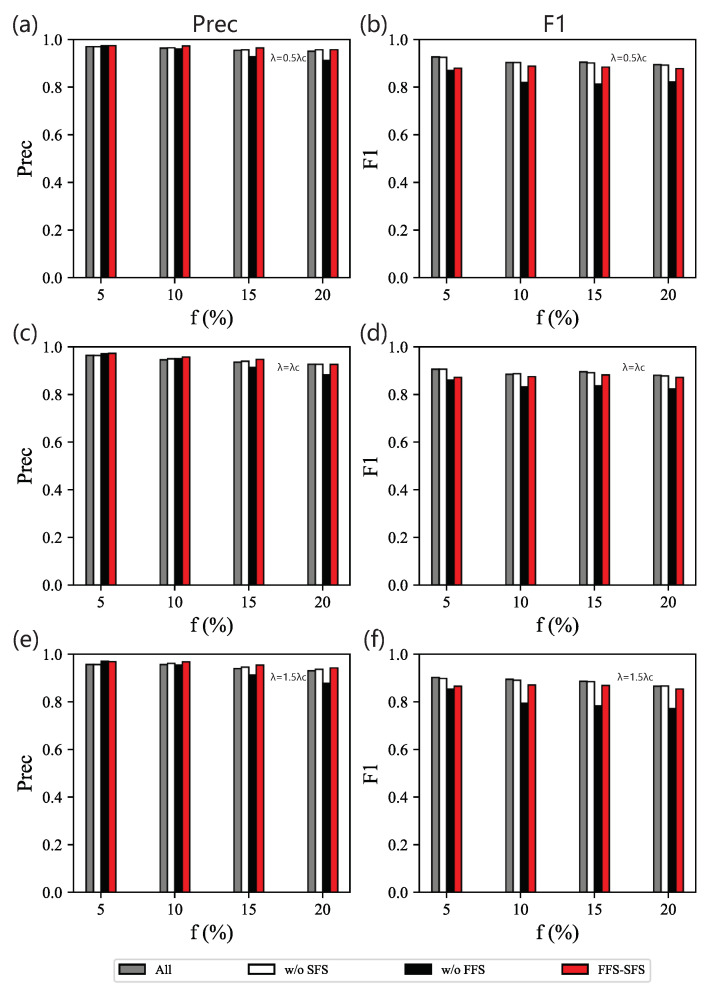
Ablation experiments on BA networks. Precision (**left** column) and F1 (**right** column) of classifiers based on different feature subsets obtained by different model components on data sets for different combinations of *f* and λ: (**a**,**b**) $\lambda =0.5\lambda_{c}$; (**c**,**d**) $\lambda =\lambda_{c}$; (**e**,**f**) $\lambda =1.5\lambda_{c}$.

When $\lambda =0.5\lambda_{c}$, the performance of FFS is similar to All, while the feature subset selected has fewer dimensions, as shown in Figure 6a,b. This shows that SFS (our method without FFS) with only the secondary selection performs badly. The feature subset selected by SFS includes two-hop, clusteringcoefficient, and betweenness for different *f*. The weakness of SFS is that it prefers to select clusteringcoefficient when clustering features on the full set, while the performance shows that combinations with clusteringcoefficient are less useful in identifying influential spreaders. Actually, clusteringcoefficient has a low correlation with the spreading process. Conversely, our method selects fewer features than the one without SFS but keeps a similar performance for most spreading data with different *f*. When $\lambda =\lambda_{c}$ (Figure 6c,d) or $\lambda =1.5\lambda_{c}$ (Figure 6e,f), the results are very similar.

Therefore, we obtain the optimal combination of centralities in BA networks using our feature selection method. When the data set is not extremely imbalanced (f=10%, 15%, or 20%), the combination of two-hop and PageRank ($\lambda =0.5\lambda_{c}$) or the combination of two-hop and degree ($\lambda =1.5\lambda_{c}$) are able to identify the top influencers in BA networks. Here, we have to note that this is a natural result since degree and PageRank have a strong linear correlation. When $\lambda =\lambda_{c}$, our feature selection method takes the combination of one-hop, two-hop, and PageRank to classify spreaders. This indicates that for $\lambda =\lambda_{c}$, more knowledge on nodes’ centralities would help to identify influential spreaders. When the data set is extremely imbalanced (f=5%), betweenness centrality is capable of classifying spreaders ($\lambda =0.5\lambda_{c},1.5\lambda_{c}$), and one-hop is more important ($\lambda =\lambda_{c}$).

### 3.2. Results on ER Networks and WS Networks

Different from BA networks, ER networks [39] and WS networks [40] are homogeneous networks, where their degree distributions are narrower than those in BA networks. WS networks are obtained at transitions between nearest-neighbor-coupled networks (edge reconnection probability p=0) and the ER networks (p=1). We generate the WS networks using p=0.2.

#### 3.2.1. Contrasting Experiment on ER Networks

As shown in Table 5, we obtain different feature subsets on ER networks. We analyze the number of features after feature selection. Intuitively, Imbalanced, FFS-ReliefF, FFS-Weight, and FFS-SFS can reduce the number of features to a certain extent. However, Imbalanced is unstable and fails to select features in most scenarios.

**Figure 7:**
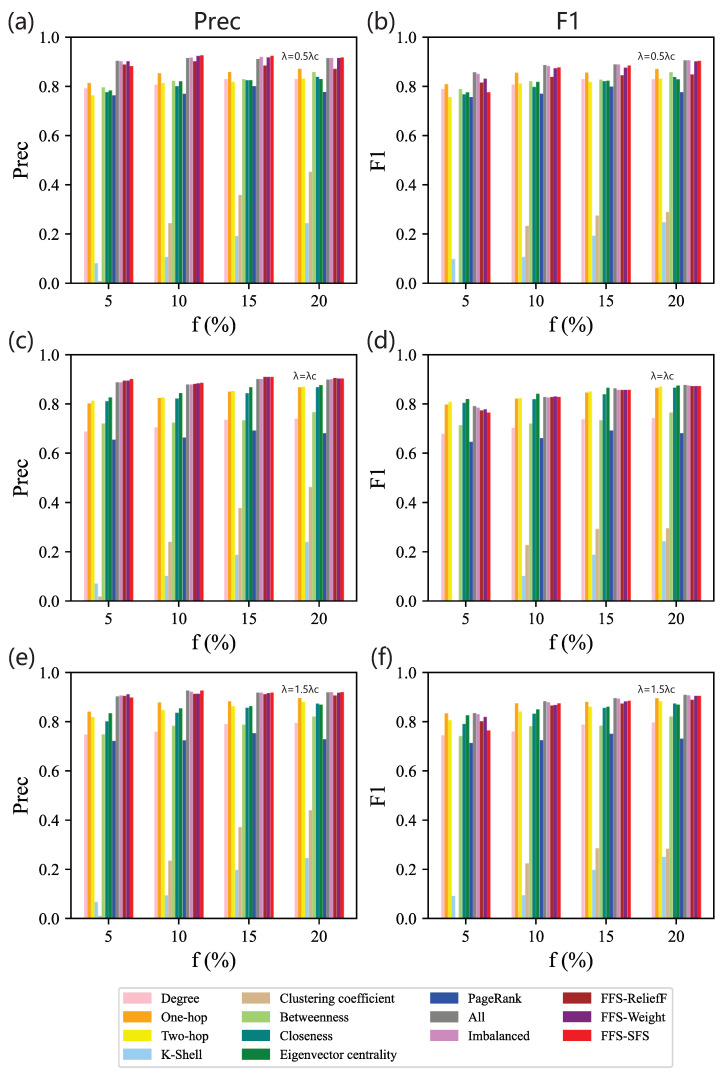
Contrasting experiments on ER networks. Precision (**left** column) and F1 (**right** column) of classifiers based on different feature subsets obtained by 13 methods on data sets for different combinations of *f* and λ: (**a**,**b**) $\lambda =0.5\lambda_{c}$; (**c**,**d**) $\lambda =\lambda_{c}$; (**e**,**f**) $\lambda =1.5\lambda_{c}$.

Figure 7 shows the Precision and F1 scores of different feature subsets obtained by the baseline methods and our method. As shown in Figure 7a, when $\lambda =0.5\lambda_{c}$, feature combination has better Precision than a single centrality, and our method improves Precision for most values of *f*. But in Figure 7b, when f>5% (f=10%,15%,20%), F1 score of one-hop surpasses FFS-ReliefF. This is because the feature subset selected by FFS-ReliefF is the combination of degree and PageRank. Since there is a strong linear correlation between these two centralities, the advantage of feature combination is not obvious. Moreover, one-hop performs better than both of them. We also obtain that the greatest gap in F1 score between our method and the baseline shrinks to 1%, while it is 2% in BA networks. This reflects that for identifying top spreaders in ER networks, even though the size of feature set increases, i.e., it is four times larger than the feature combination selected by our method, supplementary knowledge plays a minor role during identification. Overall, when λ is fixed, with the increase in *f*, Precision and F1 generally increase. This is because in ER networks, the degree distribution follows a Poisson distribution. As *f* increases, the differences in topology information between different categories become more evident. With the increase in *f*, more and wider structural knowledge is used in the training process, resulting in higher performance.

When $\lambda =\lambda_{c}$, we find that although the feature subsets selected by the five feature combination methods are distinct, their performances are remarkably similar, as shown in Figure 7c,d. This reveals that with more features, F1 score cannot obviously improve, and the feature space of each method is similar. Moreover, F1 score of eigenvectorcentrality is higher than most feature combinations across the range of *f*, so eigenvectorcentrality plays a crucial role when $\lambda =\lambda_{c}$.

When $\lambda =1.5\lambda_{c}$, we also find similar rules as the result for $\lambda =0.5\lambda_{c}$; that is, one-hop performs better than FFS-ReliefF, as shown in Figure 7e,f. Classifiers based on our feature selection method still show high Precision and competitive F1 compared with full feature sets. Similar to the results in BA networks, as shown in Figure 7, across the whole range of λ, when f=5%, hindered by undersampling, the impact of our method on enhancing effectiveness is limited.

#### 3.2.2. Ablation Experiment on ER Networks

Table 6 shows the results of the ablation experiment on ER networks. FFS can adapt to different associations of λ and *f* and reduce the number of features. The mechanism of SFS determines smaller feature space dimensions. We also find that FFS selects fewer features for ER networks than BA networks, especially when $\lambda =\lambda_{c}$. This reveals that some features such as eigenvectorcentrality are more dominant than others in ER networks.

**Figure 8:**
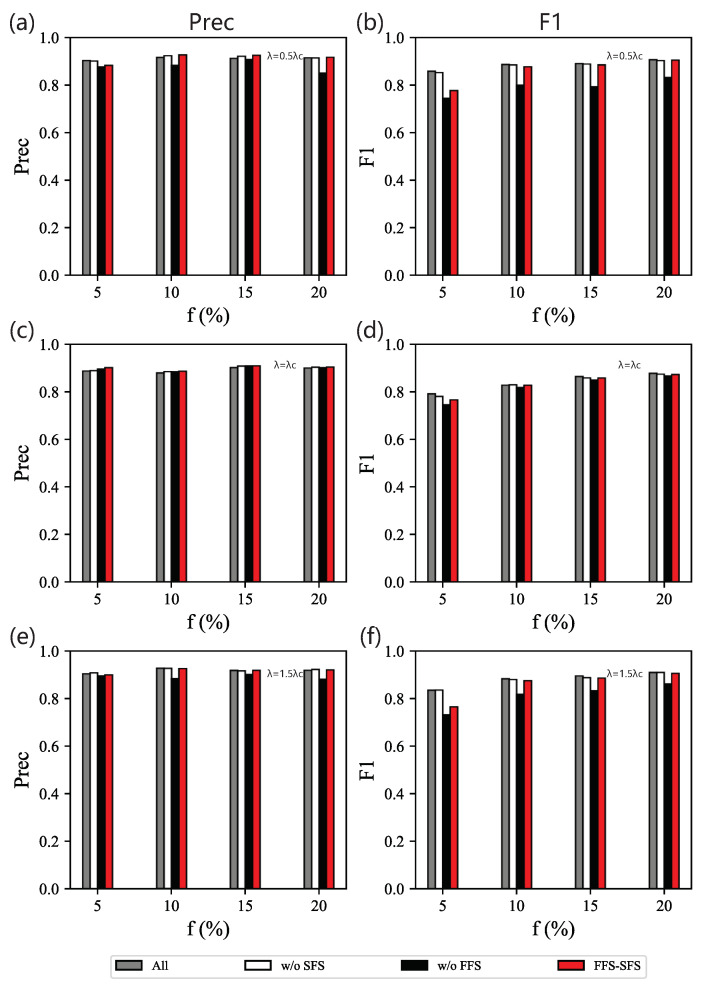
Ablation experiments on ER networks. Precision (**left** column) and F1 (**right** column) of classifiers based on different feature subsets obtained by different model components on data sets for different combinations of *f* and λ: (**a**,**b**) $\lambda =0.5\lambda_{c}$; (**c**,**d**) $\lambda =\lambda_{c}$; (**e**,**f**) $\lambda =1.5\lambda_{c}$.

When $\lambda =0.5\lambda_{c}$, as shown in Figure 8a,b, except for SFS, the performances of the other methods are comparable when f>5% (f=10%,15%,20%). Among these methods, only SFS also introduces clusteringcoefficient in ER networks, which drags down the performance. When f=5%, FFS tends to retain full feature set in most experiments, resulting in the follow-up SFS of our method inevitably choosing representative but poorly performing features, for example, clusteringcoefficient, which has weak linear relationship with other centralities.

When $\lambda =\lambda_{c}$, as shown in Figure 8c,d, F1 score is lower than when $\lambda =0.5\lambda_{c}$ for all the values of *f* we testified, and all methods perform similarly to all feature sets. The reason is that eigenvectorcentrality is an element of their feature combination and plays a fundamental role in identifying influencers. As shown in Figure 8e,f, when $\lambda =1.5\lambda_{c}$, the result is very close to that for λ=0.5λ.

In ER networks, in summary, when $\lambda =0.5\lambda_{c}$ and f>5%, our feature selection method takes the combination of degree and one-hop as key factors for identifying influential spreaders. When $\lambda =1.5\lambda_{c}$, combinations with degree are also helpful. At $\lambda =\lambda_{c}$, since the influence of the spreaders is very ambiguous to measure, to our surprise, the performance of eigenvectorcentrality is better than all the feature combinations we tested.

#### 3.2.3. Results on WS Networks

**Figure 9:**
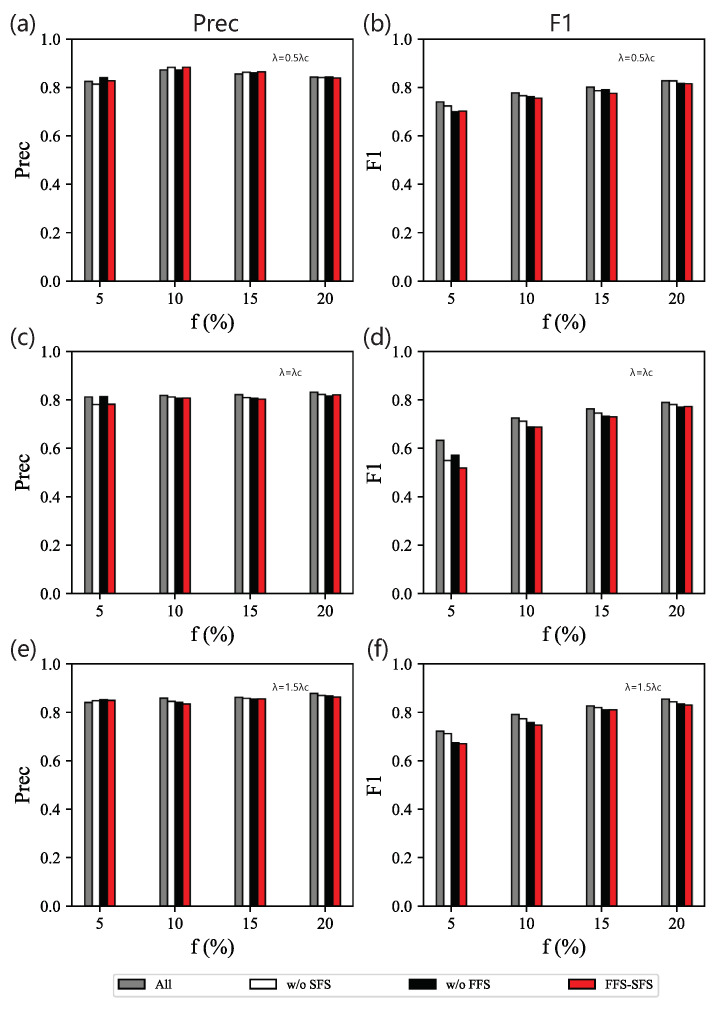
Ablation experiments on WS networks. Precision (**left** column) and F1 (**right** column) of classifiers based on different feature subsets obtained by different model components on data sets for different combinations of *f* and λ: (**a**,**b**) $\lambda =0.5\lambda_{c}$; (**c**,**d**) $\lambda =\lambda_{c}$; (**e**,**f**) $\lambda =1.5\lambda_{c}$.

**Figure 10:**
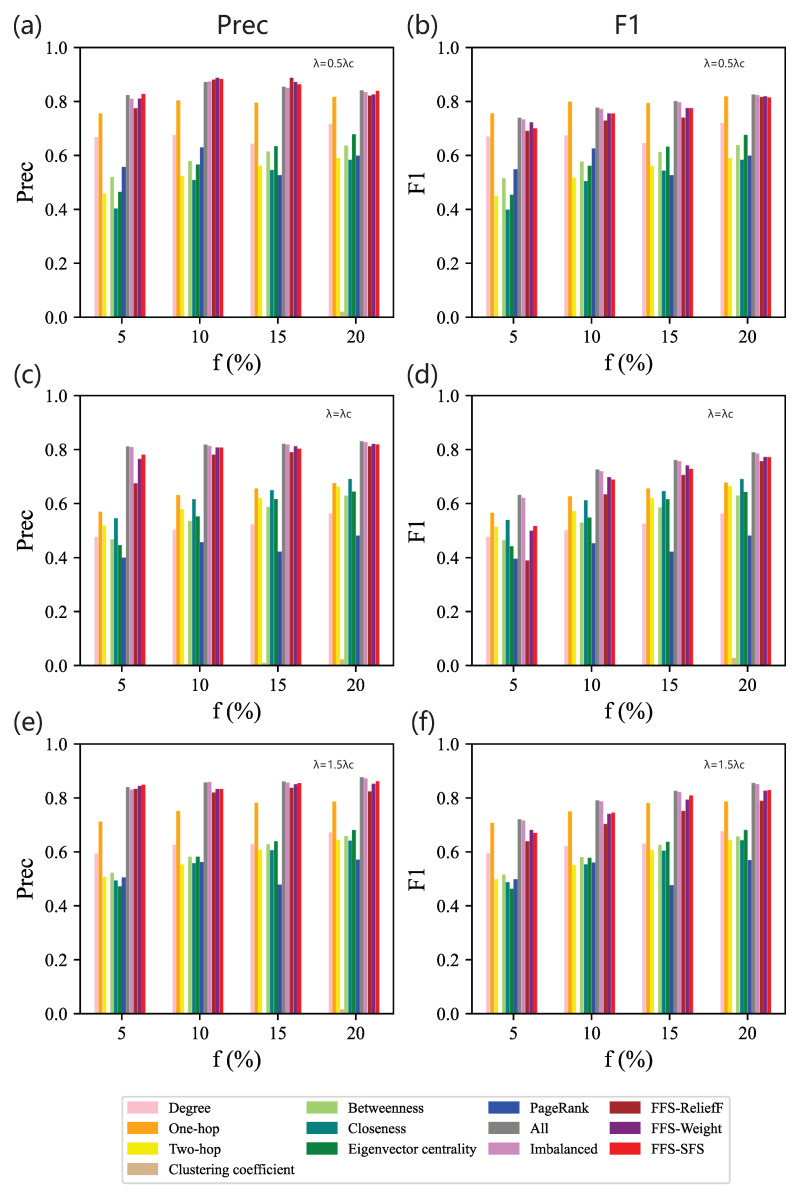
Contrasting experiments on WS networks. Precision (**left** column) and F1 (**right** column) of classifiers based on different feature subsets obtained by 13 methods on data sets for different combinations of *f* and λ: (**a**,**b**) $\lambda =0.5\lambda_{c}$; (**c**,**d**) $\lambda =\lambda_{c}$; (**e**,**f**) $\lambda =1.5\lambda_{c}$.

Figure 9 illustrates the results of the ablation experiment, and Figure 10 shows the performance of contrasting experiments on WS networks. From Figure 9 and Figure 10, we find that despite adopting the full feature set, the performance of classifiers is unsatisfactory, much lower than the performance of classifiers for BA networks or ER networks. Especially for $\lambda =\lambda_{c}$, where noise occurs during the process of disease dynamics, classifiers for WS networks perform worse. Therefore, the feature selection method is not applicable for WS networks, which requires more structural knowledge.

### 3.3. Results on Real-World Networks

To explore the similarity between the optimal centralities of real-world networks and synthetic networks, we apply our feature selection methods on four real-world networks, i.e., the musical collaboration network, Jazz [41]; the email interchange network of the university, Email [42]; the airline network, USairport [43]; and the online network of secure information interchange, Pretty Good Privacy [44]. Table 7 shows the statistical indicators of these real-world networks. For the convenience of statistics, we use the Venn diagram to obtain the most frequently selected features for each effective infection rate.

**Figure 11:**
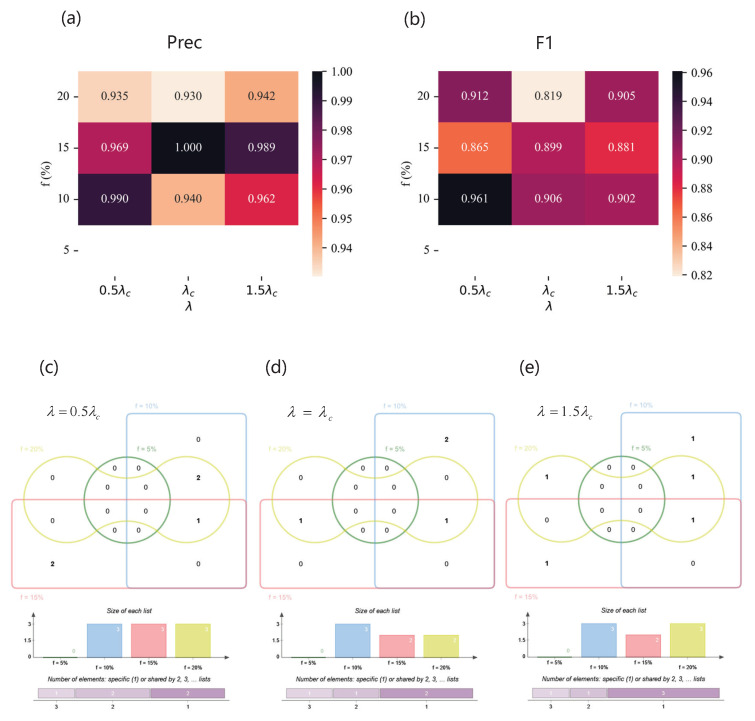
The results of the Jazz network. (**a**,**b**) Precision (**upper left**) and F1 (**upper right**) of classifiers based on different feature subsets under different combinations of λ and *f*. (**c**–**e**) Venn diagrams of feature subsets versus λ. The top of each Venn diagram: four feature subsets corresponding to different label proportions *f* for each λ, that is, f=5% (green), f=10% (blue), f=15% (red), and f=20% (yellow), where the number in the box represents the amount of features of each area. The middle of each Venn diagram: the size of each feature subset. The bottom of each Venn diagram: the number of features which are specific to one *f* or shared by multiple *f*. (**c**) When $\lambda =0.5\lambda_{c}$, the feature subsets are $\left\{K_{2sum},C,EC\right\}$ (f=10%), $\left\{K_{sum},EC,PR\right\}$ (f=15%), and $\left\{K_{2sum},C,EC\right\}$ (f=20%); (**d**) when $\lambda =\lambda_{c}$, the feature subsets are $\left\{K_{2sum},C,EC\right\}$ (f=10%), $\left\{K_{sum},EC\right\}$ (f=15%), and $\left\{K_{sum},EC\right\}$ (f=20%); (**e**) when $\lambda =1.5\lambda_{c}$, the feature subsets are $\left\{K_{2sum},C,EC\right\}$ (f=10%), $\left\{K_{sum},EC\right\}$ (f=15%), and {K,C,EC} (f=20%).

As for the Jazz network, Figure 11 illustrates the performance of the SVM based on feature subset selected by our method FFS-SFS and the Venn diagram of feature subsets of different spreading parameter combination. Figure 11a,b show Precision and F1 of the classifiers. When the imbalanced ratio is very high, i.e., f=5%, our method cannot work because of the insufficient sampling quantity of the Jazz dataset. However, despite being at the threshold of infection dynamics, the high performance shows that our model is able to select helpful feature subsets. Grouping by effective infection rate and intersecting feature combinations selected by our method under different *f*, we obtain the relatively important features of a specific propagation scenario. As shown in Figure 11c, when $\lambda =0.5\lambda_{c}$, eigenvectorcentrality is a key element of a feature subset, which covers three spreading parameter combinations, while two-hop and clusteringcoefficient cover two. As shown in Figure 11d,f, for $\lambda =\lambda_{c}$ and $\lambda =1.5\lambda_{c}$, eigenvectorcentrality also plays a important role in identification, followed by one-hop for $\lambda =\lambda_{c}$ and clusteringcoefficient for $\lambda =1.5\lambda_{c}$.

**Figure 12:**
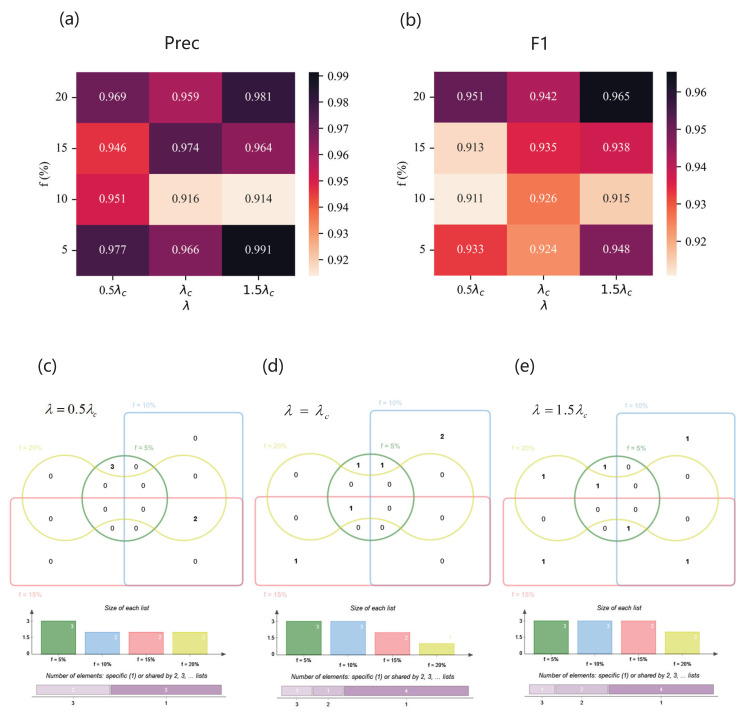
The results of the Email network. (**a**,**b**) Precision (**upper left**) and F1 (**upper right**) of classifiers based on different feature subsets under different combinations of λ and *f*. (**c**–**e**) Venn diagrams of feature subsets versus λ. The top of each Venn diagram: four feature subsets corresponding to different label proportions *f* for each λ, that is, f=5% (green), f=10% (blue), f=15% (red), and f=20% (yellow), where the number in the box represents the amount of features of each area. The middle of each Venn diagram: the size of each feature subset. The bottom of each Venn diagram: the number of features which are specific to one *f* or shared by multiple *f*. (**c**) When $\lambda =0.5\lambda_{c}$, the feature subsets are $\left\{K_{2sum},C,EC\right\}$ (f=5%), $\left\{K,K_{sum}\right\}$ (f=10%), $\left\{K,K_{sum}\right\}$ (f=15%), and $\left\{K,K_{sum}\right\}$ (f=20%); (**d**) when $\lambda =\lambda_{c}$, the feature subsets are $\left\{K_{2sum},C,EC\right\}$ (f=5%), {KS,C,B} (f=10%), $\left\{K_{sum},EC\right\}$ (f=15%), and {EC} (f=20%); (**e**) when $\lambda =1.5\lambda_{c}$, the feature subsets are $\left\{K_{2sum},C,EC\right\}$ (f=5%), {KS,C,B} (f=10%), {KS,C,PR} (f=15%), and {K,EC} (f=20%).

**Figure 13:**
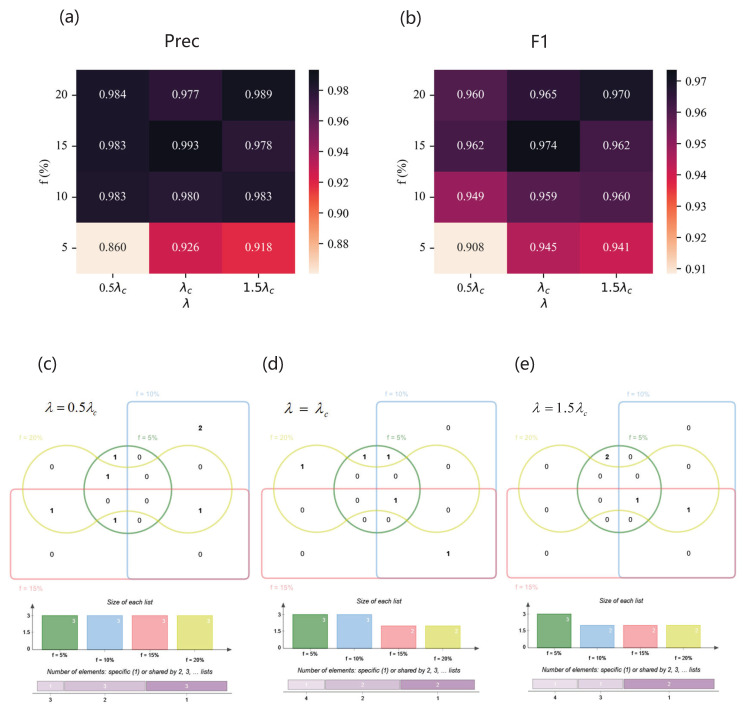
The results of the USairport network. (**a**,**b**) Precision (**upper left**) and F1 (**upper right**) of classifiers based on different feature subsets under different combinations of λ and *f*. (**c**–**e**) Venn diagrams of feature subsets versus λ. The top of each Venn diagram: four feature subsets corresponding to different label proportions *f* for each λ, that is, f=5% (green), f=10% (blue), f=15% (red), and f=20% (yellow), where the number in the box represents the amount of features of each area. The middle of each Venn diagram: the size of each feature subset. The bottom of each Venn diagram: number of features which are specific to one *f* or shared by multiple *f*. (**c**) When $\lambda =0.5\lambda_{c}$, the feature subsets are $\left\{K_{2sum},KS,B\right\}$ (f=5%), $\left\{K,K_{sum},C\right\}$ (f=10%), {K,B,EC} (f=15%), and {K,KS,EC} (f=20%); (**d**) when $\lambda =\lambda_{c}$, the feature subsets are $\left\{K,K_{sum},C\right\}$ (f=5%), $\left\{K,K_{sum},KS\right\}$ (f=10%), {K,KS} (f=15%), and {K,CC} (f=20%); (**e**) when $\lambda =1.5\lambda_{c}$, the feature subsets are $\left\{K,K_{sum},C\right\}$ (f=5%), {K,KS} (f=10%), {K,KS} (f=15%), and {K,KS} (f=20%).

**Figure 14:**
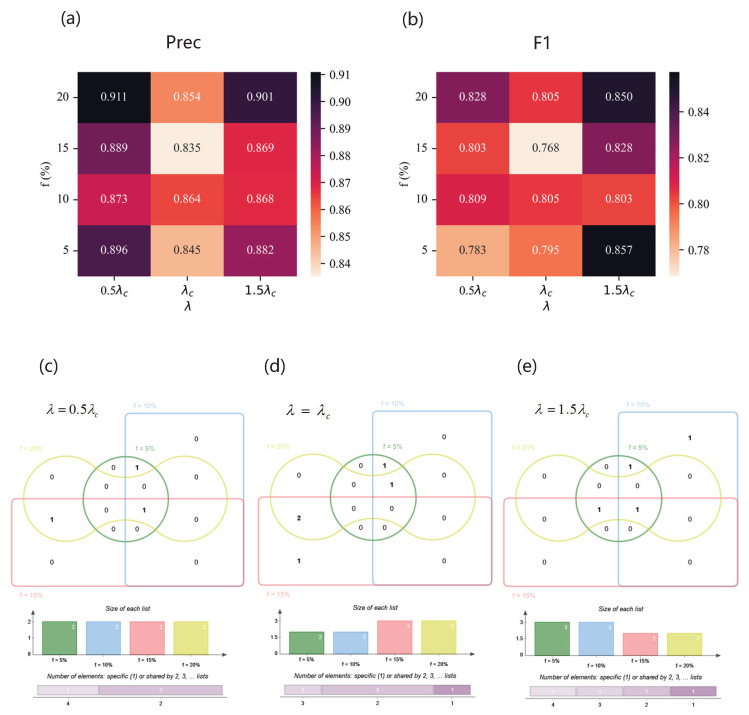
The results of the Pretty Good Privacy network. (**a**,**b**) Precision (**upper left**) and F1 (**upper right**) of classifiers based on different feature subsets under different combinations of λ and *f*. (**c**–**e**) Venn diagrams of feature subsets versus λ. The top of each Venn diagram: four feature subsets corresponding to different label proportions *f* for each λ, that is, f=5% (green), f=10% (blue), f=15% (red), and f=20% (yellow), where the number in the box represents the amount of features of each area. The middle of each Venn diagram: the size of each feature subset. The bottom of each Venn diagram: number of features which are specific to one *f* or shared by multiple *f*. (**c**) When $\lambda =0.5\lambda_{c}$, the feature subsets are $\left\{K_{2sum},KS\right\}$ (f=5%), $\left\{K_{2sum},KS\right\}$ (f=10%), $\left\{K,K_{2sum}\right\}$ (f=15%), and $\left\{K,K_{2sum}\right\}$ (f=20%); (**d**) when $\lambda =\lambda_{c}$, the feature subsets are $\left\{K_{2sum},KS\right\}$ (f=5%), $\left\{K_{2sum},KS\right\}$ (f=10%), {CC,EC,PR} (f=15%), and {KS,CC,PR} (f=20%); (**e**) when $\lambda =1.5\lambda_{c}$, the feature subsets are $\left\{K_{2sum},KS,CC\right\}$ (f=5%), {KS,B,CC} (f=10%), $\left\{K_{2sum},KS\right\}$ (f=15%), and $\left\{K_{2sum},KS\right\}$ (f=20%).

As for Email network, the performance of the classifier is shown in Figure 12. Influenced by handmade labels, the result is fluctuating, but all the scores reach over 0.91 with our method. When $\lambda =1.5\lambda_{c}$ and f=20%, Precision is at the maximum. And the best F1 can be achieved in the situation of $\lambda =1.5\lambda_{c}$ and f=5%. When $\lambda =0.5\lambda_{c}$, degree and one-hop span three spreading parameter combinations. When $\lambda =\lambda_{c}$, eigenvectorcentrality encompasses three spreading parameter combinations. When $\lambda =1.5\lambda_{c}$, clusteringcoefficient extends over three spreading parameter combinations. As for the USairport network, the result is shown in Figure 13. On the whole, with the increase in λ and *f*, the performance of the classifier roughly becomes higher. Especially when $\lambda =\lambda_{c}$ and f=15%, Precision and F1 are both at their maximum. Degree covers most spreading parameter combinations. As for the Pretty Good Privacy network, the results are shown in Figure 14. When $\lambda =0.5\lambda_{c}$ and f=20%, the optimal Precision is achieved, and when $\lambda =1.5\lambda_{c}$ and f=5%, the highest F1 is attained. Two-hop and K−Shell play crucial roles in the majority of spreading parameter combinations.

According to the results from real-world networks, the features selected by our method have substantial overlap with the ones obtained on synthetic networks, indicating that synthetic networks describe fundamental properties of real-world networks. And according to the above results, neighborhood-based centralities, including degree, one-hop, two-hop, and K−Shell, are always selected, which is due to the fact that the result of disease spreading depends on the neighborhood of the central infected spreader. Moreover, we also find that the size of training data sets affects the performance of the classifier, i.e., the performance of the SVM on the Email or USairport networks is better than Jazz (a small-scale network) or Pretty Good Privacy (a large-scale network).

## 4. Conclusions

When using machine learning methods to identify influential spreaders, the selection of centrality metrics remains unresolved. Based on the classification of imbalanced data, we propose a two-phase feature selection method named FFS-SFS to obtain the optimal combination of centralities for the identification task. According to the experimental results of three representative synthetic networks and four real-world networks, our method can effectively reduce the dimensions, and the features selected have better classification performance than most baseline methods. Our supervised method finds that, for synthetic networks, the combination of centralities with a two-hop neighborhood is deemed optimal for the BA network, while the combination of centralities with degree matches the ER network. However, for the WS network, due to the higher homogeneity among the nodes, the identification task requires more network topology information. We also applied our feature selection method to various real-world networks and discovered an overlap in the results between synthetic and real-world networks. Our results reveal that the neighborhood-based centralities, such as degree and two-hop neighborhood, play crucial roles in identifying influential spreaders across a wide range of networks.

## 5. Discussion

There are still some limitations to our work. Our supervised method only focuses on the topological properties of the underlying network in the spreading process. However, in the real world, non-topological attributes like age, i.e., elderly people and young children are more susceptible to infection, also affect the influence of each spreader. In future work, we will enhance the generalizability of our method by incorporating more non-topological attributes on real-world data [45]. For the underlying network, when generating the dataset for large real-world networks, we encounter expensive computational problems such as calculating betweenness centrality. We can explore the use of subgraph sampling techniques to improve our methods in the future. Except for the limitations of the underlying network, in terms of network dynamics, we only apply the SIR model to simulate the influence of each node of the spread of the epidemic. However, disease propagation in real life is complex. For example, before symptoms become apparent, most diseases undergo an incubation period, so that other models similar to SIR, like SEIR [5], will be considered in the future, or we will further explore model-free simulation [46]. Moreover, although the SIR model is also applicable to the field of rumor spreading, etc., it cannot fully capture properties of some spreading dynamics such as behavior spreading [47]. In the future, we will explore a more general method to uncover the characteristics of different spreading processes effectively.

## Figures and Tables

**Table 1:** Selected centralities. In the formulas, *A* is an adjacency matrix, $A_{ij}=1$ if node *i* has a relationship with node *j*, and $A_{ij}=0$ otherwise; $\Omega_{1}\left(i\right)$ is the one-hop neighbor set of node *i*; $\Omega_{2}\left(i\right)$ is the two-hop neighbor set of node *i*; E(i) is the amount of edges among the elements of $\Omega_{1}\left(i\right)$; $g_{st}$ is the number of shortest paths between node *s* and node *t*; $g_{st}\left(i\right)$ is the number of shortest paths between node *s* and node *t* passing node *i* as a bridge; $d_{ij}$ is the distance between node *i* and node *j*; $\lambda_{i}$ is the leading eigenvalue of matrix *A*; and α and β are both hyperparameters.

Centrality	Definition	Formula
*K*	Counting the number of one-hop neighbors of a node	$K\left(i\right)=\sum_{j\neq i}A_{ij}$
$K_{sum}$	Counting the degrees of one-hop neighbors of a node	$K_{sum}\left(i\right)=\sum_{j\in \Omega_{1}\left(i\right)}K\left(j\right)$
$K_{2sum}$	Counting the degrees of two-hop neighbors of a node	$K_{2sum}\left(i\right)=\sum_{j\in \Omega_{2}\left(i\right)}K\left(j\right)$
KS	Resulting from K-shell decomposition [10]	-
*C*	Measuring the fraction of triangles around the node	$C\left(i\right)=\frac{2E(i)}{K(i)(K(i)-1)}$
*B*	Measuring the ability of a node when regarded as the bridge between pairs of nodes	$B\left(i\right)=\sum_{s\neq i\neq t}=\frac{g_{st}\left(i\right)}{g_{st}}$
CC	Averaging the shortest path lengths to other nodes	$CC\left(i\right)=\frac{N-1}{\sum_{j=1,j\neq i}^{N}d_{ij}}$
EC	Combining with the centrality of its neighbors to obtain the result	$EC\left(i\right)=\lambda_{i}^{-1}\sum_{j}A_{ij}EC\left(j\right)$
PR	The variant of eigenvector centrality	$PR\left(i\right)=\alpha \sum_{j}A_{ij}\frac{PR(j)}{K(j)}+\beta$

**Table 2:** A sample of the data set in a BA network with N=1000, 〈k〉=6.

Node	*K*	$K_{\mathrm{sum}}$	$K_{2\mathrm{sum}}$	KS	*C*	*B*	CC	EC	PR	Label
1	6	52	568	3	0	793.44	0.2818	0.0124	0.0010	−1
2	4	55	441	3	0.1667	322.06	0.2733	0.0108	0.0007	−1
3	4	115	1060	3	0	463.69	0.3091	0.0278	0.0006	1
4	6	58	545	3	0	642.25	0.2807	0.0101	0.0010	−1
…	…	…	…	…	…	…	…	…	…	
1000	13	135	1268	3	0.0128	2592.87	0.3175	0.0332	0.0021	1

**Table 3:** Contrasting experiment. Size of feature subsets on BA networks based on different feature selection methods.

Method	$\lambda =0.5\lambda_{c}$					$\lambda =\lambda_{c}$					$\lambda =1.5\lambda_{c}$			
	f=5%	f=10%	f=15%	f=20%		f=5%	f=10%	f=15%	f=20%		f=5%	f=10%	f=15%	f=20%
All	8	8	8	8		8	8	8	8		8	8	8	8
Imbalanced	8	8	7	8		8	8	8	8		8	8	8	8
FFS-ReliefF	3	3	2	2		3	3	3	3		3	2	2	2
FFS-Weight	3	3	2	2		3	3	3	3		3	3	2	2
FFS-SFS	3	3	2	2		3	3	3	3		3	2	2	2
Selected by FFS-SFS	{two-hop, clustering, betweenness}	{one-hop, two-hop, PR}	{two-hop, PR}	{two-hop, PR}		{two-hop, clustering, betweenness}	{one-hop, two-hop, PR}	{one-hop, two-hop, PR}	{one-hop, two-hop, PR}		{two-hop, clustering, betweenness}	{degree, two-hop}	{degree, two-hop}	{degree, two-hop}

**Table 4:** Ablation experiment. Size of feature subsets on BA networks based on different feature selection methods.

Method	$\lambda =0.5\lambda_{c}$					$\lambda =\lambda_{c}$					$\lambda =1.5\lambda_{c}$			
	f=5%	f=10%	f=15%	f=20%		f=5%	f=10%	f=15%	f=20%		f=5%	f=10%	f=15%	f=20%
w/o SFS	8	5	5	3		8	6	5	6		8	5	5	4
w/o FFS	3	3	3	3		3	3	3	3		3	3	3	3
FFS-SFS	3	3	2	2		3	3	3	3		3	2	2	2
Selected by FFS-SFS	{two-hop, clustering, betweenness}	{one-hop, two-hop, PR}	{two-hop, PR}	{two-hop, PR}		{two-hop, clustering, betweenness}	{one-hop, two-hop, PR}	{one-hop, two-hop, PR}	{one-hop, two-hop, PR}		{two-hop, clustering, betweenness}	{degree, two-hop}	{degree, two-hop}	{degree, two-hop}

**Table 5:** Contrasting experiment. Size of feature subsets in ER networks based on different feature selection methods.

Method	$\lambda =0.5\lambda_{c}$					$\lambda =\lambda_{c}$					$\lambda =1.5\lambda_{c}$			
	f=5%	f=10%	f=15%	f=20%		f=5%	f=10%	f=15%	f=20%		f=5%	f=10%	f=15%	f=20%
All	9	9	9	9		9	9	9	9		9	9	9	9
Imbalanced	9	9	9	2		9	2	9	3		9	9	9	3
FFS-ReliefF	3	2	2	2		3	2	2	2		3	3	3	3
FFS-Weight	3	2	2	2		3	1	1	2		3	3	3	3
FFS-SFS	3	2	2	2		3	2	1	2		3	3	3	2
Selected by FFS-SFS	{k-shell, clustering, betweenness}	{degree, one-hop}	{degree, one-hop}	{degree, one-hop}		{two-hop, k-shell, clustering}	{two-hop, EC}	{EC}	{two-hop, EC}		{k-shell, clustering, EC}	{degree, one-hop, EC}	{degree, two-hop, closeness}	{degree, two-hop}

**Table 6:** Ablation experiment. Size of feature subsets in ER networks based on different feature selection methods.

Method	$\lambda =0.5\lambda_{c}$					$\lambda =\lambda_{c}$					$\lambda =1.5\lambda_{c}$			
	f=5%	f=10%	f=15%	f=20%		f=5%	f=10%	f=15%	f=20%		f=5%	f=10%	f=15%	f=20%
w/o SFS	9	3	6	2		9	1	1	1		9	4	4	4
w/o FFS	3	3	3	3		3	3	3	3		3	3	3	3
FFS-SFS	3	2	2	2		3	2	1	2		3	3	3	2
Selected by FFS-SFS	{k-shell, clustering, betweenness}	{degree, one-hop}	{degree, one-hop}	{degree, one-hop}		{two-hop, k-shell, clustering}	{two-hop, EC}	{EC}	{two-hop, EC}		{k-shell, clustering, EC}	{degree, one-hop, EC}	{degree, two-hop, closeness}	{degree, two-hop}

**Table 7:** Attributes of the real-world networks. *N* is the number of nodes and *m* is the number of edges. For each network, 〈k〉 is the average degree, $k_{max}$ is the max degree, *c* is the average clustering coefficient, and *d* is the density. All the networks are connected.

Network	*N*	*m*	〈k〉	$k_{\max}$	*c*	*d*
Jazz	198	2742	27.697	100	0.6175	0.140593
Email	986	16,687	32.751	347	0.4071	0.034363
USairport	1572	17,214	34.428	314	0.5048	0.013941
Pretty Good Privacy	10,680	24,316	4.554	205	0.2659	0.000426

## Data Availability

Not applicable.
